# A renin transcript lacking exon 1 encodes for a non-secretory intracellular renin that increases aldosterone production in transgenic rats

**DOI:** 10.1111/j.1582-4934.2008.00132.x

**Published:** 2008-08-11

**Authors:** Jörg Peters, Heike Wanka, Barbara Peters, Sigrid Hoffmann

**Affiliations:** a Department of Cardiovascular Medicine, University of GreifswaldGreifswald, Germany; b Department of Physiology, University of GreifswaldGreifswald, Germany; c *Medical Research Center, Faculty for Clinical Medicine Mannheim, University of HeidelbergHeidelberg, Germany

**Keywords:** renin-angiotensin system, cytoplasmatic renin, adrenal gland, aldosterone, transgenic rats

## Abstract

Renin transcripts lacking exon 1 and thus the signal sequence for co-translational transport to the endoplasmatic reticulum encode for a protein (exon[2-9]renin), that is confined to the cytoplasm. The function of exon(2-9)renin is currently unknown. Mitochondrial renin increases under conditions which stimulate aldosterone production. We hypothesized that exon(2-9)renin (1) is translated into a functionally active protein *in vivo*, (2) is not secreted but remains within the cytoplasm and (3) stimulates aldosterone production. To test these hypotheses we generated transgenic rats overexpressing exon(2-9)renin. Four transgenic lines were obtained expressing the transcript in various tissues including the heart and the adrenal gland. Renin was enriched particularly in the cytoplasm of transgenic rats. Renin was not elevated in plasma, indicating that exon(2-9)renin is produced but not secreted. The ratio of aldosterone to renin concentrations in plasma (PAC/PRC) was elevated in all transgenic lines except line 307, which also did not exhibit elevated cytoplasmatic renin levels in the adrenal gland (PAC/PRC in controls: 2.8±2.3; line 307: 1.9±0.8; n. s.; line 284: 5.8±1.9; *P*<0.02; line 294: 5.0±2.3; *P*<0.001; line 276: 10.3±5.1; *P*<0.001). We conclude that the exon(1A-9) renin transcript (1) is translated into a functionally active intracellular protein; (2) is targeted to the cytoplasm rather than being sorted to the secretory pathways and (3) is functionally active, regulating aldosterone production. The CX-(exon2-9)renin transgenic rat appears to be a useful model to study the role and the mechanisms of action of cytoplasmatic renin derived from exon(1A-9) transcripts.

## Introduction

The circulating renin-angiotensin system (RAS) plays a central role in the regulation of blood pressure as well as water and electrolyte balance. In the adrenal gland the RAS stimulates aldosterone production [1]. In the heart it promotes hypertrophy, apoptosis, inflammation, fibrosis, and hence cardiac failure [2–4]. In the brain the RAS is involved in central blood pressure regulation and control of volume homeostasis [5].

Renin is the key enzyme of the RAS. It cleaves angiotensin (ANG) I from its only known substrate, angiotensinogen. ANGI is cleaved by angiotensin-converting enzyme (ACE) to the effector peptide ANGII. Until recently, renin was exclusively known as a secretory glycoprotein predominantly expressed in the kidney [6]. Other tissues, such as the adrenal gland also express and secrete renin, albeit to a much lesser extend than the kidney [7].

The targeting of proteins to secretory pathways requires the co-translational transport to the endoplasmatic reticulum (ER). The transport signal for renin is encoded by a signal sequence derived from exon 1 of the renin gene. We have isolated and characterized a second renin transcript, termed exon(1A-9)-renin, which codes for a non-secretory protein [8]. Similar transcripts have been found in other species as well, including humans [9, 10]. These transcripts lack exon 1 and thus the signal for the co-translational transport to the ER. In humans one of the renin transcripts directly starts with exon 2 [10]. In rats, exon 2 of the exon(1A-9)renin transcript is preceded by a short sequence of about 80 base pairs derived from intron A [8]. This sequence is non-coding and therefore can only have regulatory functions.

The exon(1A-9)renin transcript is translated into a truncated prorenin starting at the first in-frame ATG in exon 2. The resulting protein lacks the pre-fragment of secretory renin (from now on termed exon[1-9]renin) as well as the first 10 amino acids of the conventional prorenin. Most tissues including the adrenal gland express both transcripts, whereas the kidney expresses exclusively the exon(1-9)renin transcript and the heart expresses exclusively the exon(1A-9)renin transcript [11]. In the heart, exon(1A-9)renin transcript levels rose after myocardial infarction [11], indicating that exon(2-9)renin may play a role in cardiac pathology or repair processes.

The functions of the truncated non-secretory exon(2-9)renin transcript-derived prorenin are currently unknown. In the adrenal cortex renin proteins are found not only within secretory vesicles but also within mitochondria, where important steps of aldosterone production take place [12, 13]. Mitochondrial renin must be derived from the exon(1A-9)renin transcript, since only this transcript renders a protein that is located in the cytosol and therefore available for mitochondrial import. In support of this view, we have demonstrated that exon(2-9)renin but not exon(1-9)renin or active renin is actively imported into isolated adrenal mitochondria *in vitro*[8]. Furthermore, stimulation of aldosterone production by bilateral nephrectomy is associated with increased levels of mitochondrial renin [13], indicating a role for exon(2-9)renin in aldosteroneproduction.

The aims of the present study were to test the hypothesis that (1) the exon(2-9)renin exists *in vivo*, (2) exon(2-9)renin is not secreted but remains within the cytoplasm and (3) overexpression of cytoplasmatic exon(2-9)renin increases aldosterone production. To this end we established a new transgenic rat model which allows to study the function of exon(2-9)renin in various tissues, such as adrenal gland, heart and brain.

## Methods

### Generation of transgenic rats (TGR)

An exon(2-9)renin DNA construct was derived from the full length rat pre-prorenin cDNA [14] as described previously [8] and cloned into pCDNA 3 (Invitrogen). A 1.12-kb exon(2-9) *BamHI/Hind III* fragment was ligated 3′ to the hybrid CX-promoter [15, 16]. Exon(2-9)renin cDNA was placed into an *EcoR1* site in front of a rabbit β-globin polyadenylation signal which terminates the construct. The 3.5-kb CX-exon(2-9)renin transgene was excised by *Sal1/HindIII* from the vector and gel purified. The transgenic rat lines were produced by microinjecting the purified DNA fragment into the pro-nuclei of fertilized oocytes of Sprague-Dawley rats as described previously [17]. Transgenic pups were detected by Southern blotting of *EcoR1*-digested tail DNA. A [^32^P]-labeled 1.4-kb *Apa1*fragment of the transgenic construct, which consisted of 340 bp of the promoter region and 1097 bp of the exon(2-9)renin cDNA was used as a probe. Starting with generation F2, genotyping was performed by PCR using 5′-TGTTACCTCCCCCGTGGTCCTC-3′ as a sense primer corresponding to positions 422-443 of the renin cDNA and 5′-CGGTGACCTCTCCAAAGGTCTGTG-3′ as an antisense primer corresponding to positions 721-744 of the renin cDNA. For each transgenic line the non-transgenic littermates were used as controls with normal renin expression.

### Analysis of renin transcript levels

Rats were decapitated under ether anaesthesia. Tissues were quickly removed, frozen in liquid nitrogen and stored at (80°C until further processing. Total RNA was extracted by means of guanidium isothiocyanate-caesium chloride centrifugation (TRIzol reagent [Life Technologies BRL]). To detect transgene expression, specific primers were used which did not recognize endogenous renin: the forward primer was located in exon B of the hybrid CX promoter [15] (5′ CTGGGCAACGTGCTGGTT 3′); the backward primer was located in the renin gene, position 721-744 of the cDNA sequence (5′ CGGTGACCTCTC CAAAGGTCTGTG 3′).

### Determination of prorenin, renin and aldosterone and electrolyte concentrations

Plasma prorenin, renin and aldosterone levels were determined as described previously from ethylenediaminetetraacetic acid (EDTA) blood obtained from the retro-orbital plexus after light ether anaesthesia [18], except that ANGI determination was based on a solid phase radioimmunoassay. Sub-cellular fractions were prepared by differential centrifugation as described previously [13]. Protein, renin and prorenin levels were determined as described previously [18]. The specificity of the enzyme reaction was controlled using the renin inhibitor CH732 [13]. Serum sodium and potassium concentrations were determined using the ISE Analysator AVL 988-4 (AVL Medical Instruments, Bad Homburg, Germany).

### Cardiac histology

Hearts were preserved in 4% formalin, embedded in paraffin. Coronal slices (2–3 μm) were stained with haematoxylin eosin. Sections were investigated by light microscopy at magnifications of x 200 and x 400 (Axioplan, Zeiss) in a blinded manner by an observer who was unaware of the different study groups.

### Blood pressure measurements

Systolic blood pressure was determined by tail plethysmography in conscious trained and pre-heated wild-type (WT) rats (10 female, 9 male) and TGR (8 female, 18 male). Rats were trained on five consecutive days. Measurements were performed on three consecutive days.

### Statistical analyses

Prorenin, renin and aldosterone levels were compared between WT and TGR of the same line by t-test. Blood pressure levels in male and female rats were compared by two way analysis of variance. Data are reported as mean ± SEM. Statistical significance was accepted at *P*<0.05.

## Results

### Generation of TGR and transgene expression in various tissues

**Fig. 1:**
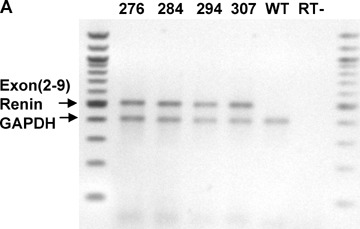

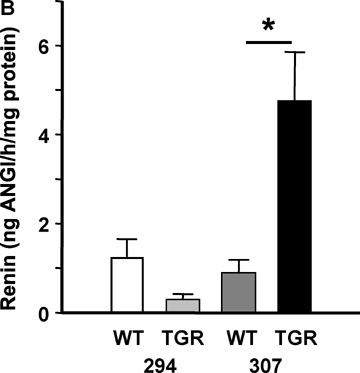
Expression of the exon(2-9)renin transgene and renin levels in the heart. (**A**): Representative blot showing transgene expression (by RT-PCR) in the hearts of all four lines of TGR and wild-type (WT) controls. (**B**): Active renin levels in total cardiac homogenates from TGR294 and TGR307 as well as their respective controls. *n***=** 6–9 per group. *: *P*<0.001 (TGR *versus*WT).

**Fig. 3:**
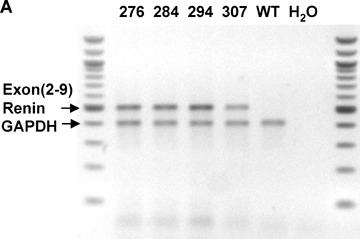

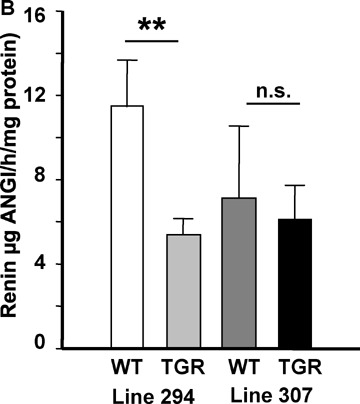

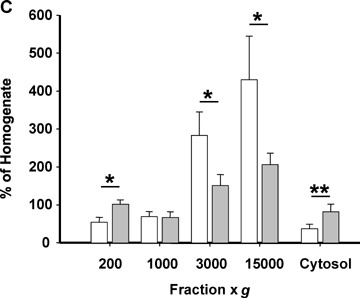
Expression of the exon(2-9)renin transgene and renin levels in the adrenal gland (**A**): Representative blot showing transgene expression in the adrenal glands of all four lines of TGR and WT controls. (**B**): Active renin levels in total adrenal gland homogenates from TGR294 and TGR307 as well as their respective controls. **: *P*<0.01 (TGR *versus*WT). (**C**): Enrichment of active renin levels in adrenal sub-cellular fractions of TGR294 (grey bars) compared with WT (open bars). *n***=** 6–9 per group; *: *P*<0.05 (TGR *versus* WT); **: *P*<0.01 (TGR *versus* WT).

Independent microinjection experiments resulted in four lines of TGR. The lines were termed TGR276, TGR284, TGR294 and TGR307. The latter two lines (TGR294 and TGR307) reproduced well, whereas reproduction was limited in TGR276 and TGR284. TGR294 and TGR307 could therefore be investigated in more detail than TGR276 and TGR284. Transgene expression was detected by means of RT-PCR in a number of tissues including kidney, adrenal gland, heart, brain, liver and spleen (Table 1). All four transgenic lines expressed the transgene in our primary target tissues heart and adrenal gland (Table 1, Figs. 1A and 3A).

**Table 1:** Expression of the exon(2-9)renin transgene in TGR276, 284, 294 and 307

	WT	Linie 276	Linie 284	Linie 294	Line 307
Kidney	-	+	+	+	+
Adrenal	-	+	+	+	+
Heart	-	+	+	+	+
Brain	-	+	+	+	+
Liver	-	(+)	-	+	-
Spleen	-	-	-	+	-

-, no expression; +, expression; (+), questionable expression

### Intracellular renin activities in the heart

**Fig. 2:**
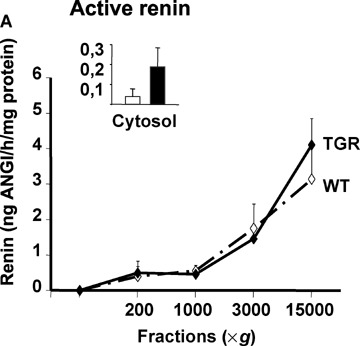

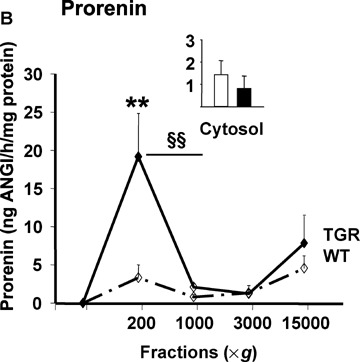

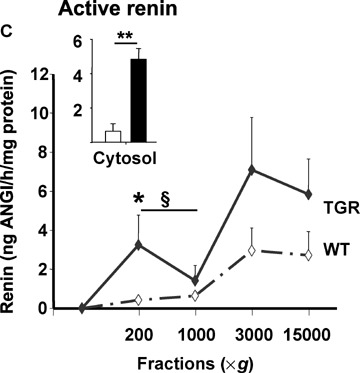

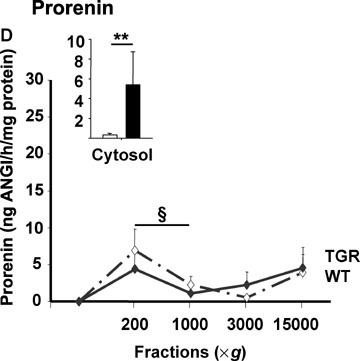
Sub-cellular distributions of active renin and prorenin in the hearts of TGR294 (**A** and **B**) and TGR307 (**C** and **D**). *n***=** 6–9 per group; §: *P*<0.05 (200 ×*g* versus 1000 ×*g*); §§: *P*<0.01 (200 ×*g* versus 1000 ×*g*); *: *P*<0.05 (TGR *versus*WT); **: *P*<0.01 (TGR *versus*WT).

Cardiac renin levels were low, approaching the detection limit in three out of the four lines investigated, including both lines of WT controls and TGR294 (Figs. 1B and 2). In TGR307, cardiac renin activity was fivefold higher than in the appropriate WT control line (Fig. 1B). More detailed analyses revealed that cardiac renin activity in TGR307 was elevated predominantly in the cytosolic fraction, but also in the 200 ×*g* fraction, representing the nuclei together with some mitochondria and cytosol (Fig. 2C). There were no differences between TGR307 and their WT controls with respect to renin activities in the low density fractions (3000 ×*g* and 15,000 ×*g*) where secretory renin is expected. Cardiac prorenin (trypsin-activatable inactive renin) levels were increased in the 200 ×*g* fraction of TGR294 (Fig. 2B) and in the cytosol of TGR307 (Fig. 2D) compared to their respective WT controls. Renin levels in the 200 ×*g* fraction of TGR307 and prorenin levels in the 200 ×*g* fractions of TGR307 and TGR294 were significantly higher than in the 1000 ×*g* fraction. Thus, there was a specific enrichment in the 200 ×*g* fraction and contamination from the fractions of lower density, 3000 ×*g* and 15,000 ×*g* (Fig. 2B–D) was excluded.

### Cardiac histology

Cardiac histology (data not shown) was normal in TGR294 and TGR307 despite overexpression of exon(2-9)renin and increased renin activities in the cytoplasm and high-density sub-cellular fraction of the latter strain.

### Intracellular renin activities in the adrenal gland

Adrenal renin activities were considerably higher than cardiac renin activities and considerably above the detection limit in all groups (Fig. 3B). TGR294 showed decreased and TGR307 normal adrenal renin activities when compared to their respective WT controls. To investigate the intracellular distribution of renin, we calculated the enrichment of renin in sub-cellular fractions normalized to renin activities in the total homogenates (Fig. 3C). There was significant enrichment of renin activities in the cytosol and the high-density fraction (200 ×*g*) of TGR294 compared with their WT controls. Furthermore, there was less enrichment of renin activities in the low density fractions (3000 ×*g*, 15,000 ×*g*) of TGR294 than in the low-density fractions of WT controls (each *P*<0.05, Fig. 3C). Renin activities were similar in sub-cellular fractions of TGR307 and their WT controls. We failed to detect prorenin in adrenal tissue in any of the groups (not shown).

### Prorenin, renin and aldosterone and electrolyte concentrations

**Fig. 4:**
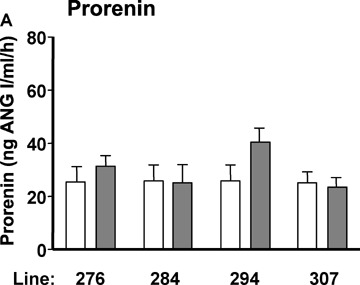

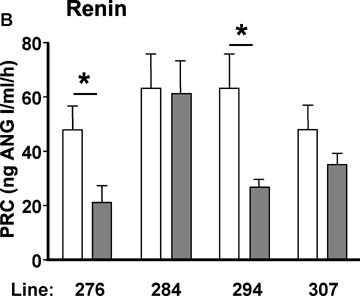

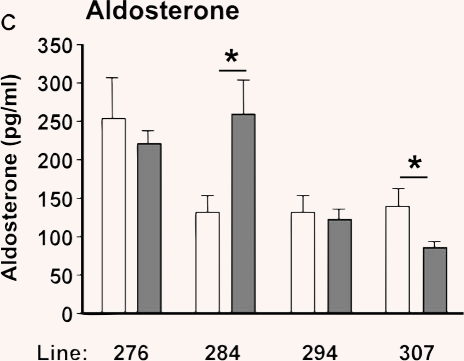

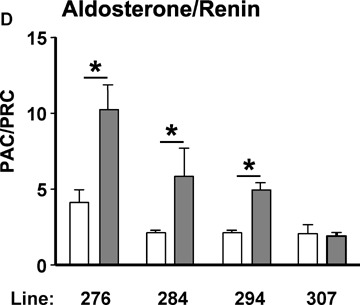
Plasma prorenin (**A**), active renin (PRC) (**B**) and aldosterone concentrations (PAC) (**C**) as well as PAC/PRC ratio (**D**) in WT (open bars) and TGR (grey bars). *n***=** 6–9 per group. *: *P*<0.02 (TGR *versus*WT).

Plasma prorenin concentrations were not different between TGR of any line and their respective WT controls (Fig. 4A). Plasma active renin concentrations in TGR were either unchanged (TGR284 and TGR207) or significantly lower than in WT controls (TGR276 and TGR294) (Fig. 4B). Plasma aldosterone concentrations were unchanged (TGR276 and TGR294), increased (TGR284) or decreased (TGR307). Despite this variability in plasma aldosterone concentrations, the ratio of plasma aldosterone concentration to plasma renin concentration was markedly elevated in all transgenic lines except TGR307 (Fig. 4D). As expected there were no changes in serum sodium and potassium levels in any of the transgenic lines (not shown).

### Systolic blood pressure

There were no statistically significant differences between male and female TGR and their age and sex-matched WT controls in any of the lines (male transgenic versuscontrols: 132±4 *versus* 132±6 mmHg; female transgenic *versus* controls: 118±11 *versus* 119±15 mmHg).

## Discussion

The present study demonstrates that (pro)renin is sorted to different intracellular compartments dependent on the presence or absence of exon 1. Without exon 1, (pro)renin remains within the cell and is not sorted to the secretory pathway. Cells transfected with a construct giving rise to an exon(1–9)renin transcript transport the encoded protein, exon(1–9)renin, to the low-density fraction and release prorenin into the medium. In contrast, cells transfected with a construct giving rise to either an exon(1A-9)renin transcript or an exon(2-9)renin transcript transport the encoded protein, exon(2-9)renin, to the high density fraction and do not release renin into the medium (J.Peters, unpublished). In the transgenic model presented here this view is supported by the fact that neither prorenin nor renin levels were increased in the circulation in CX-exon(2-9)renin TGR, despite considerable overexpression of functionally active renin in tissues. In the present study we focused on the roles of exon(2-9)renin in the heart and the adrenal gland.

### Exon(2-9)renin and the heart

The circulating RAS is known to exert pro-inflammatory and pro-fibrotic effects on the heart [2–4], whereas ACE inhibitors reduce mortality rates in patients with congestive heart failure [19]. Since the rat heart exclusively expresses exon(1A-9)renin, but not exon(1–9)renin [11] these effects are mediated by secretory exon(1–9)renin that is taken up from the circulation [20–21] and not by locally expressed exon(1–9)renin. It is presently not clear whether locally expressed intracytoplasmatic exon(1A-9)renin exerts detrimental or protective effects on the heart. Since the expression of exon(1A-9)renin is selectively up-regulated after myocardial infarction [11], intracellular renin may play a role in the pathophysiology of ischemia-related repair processes.

In the present study we observed a fivefold overexpression of exon(2-9)renin in the heart and demonstrated its localization in the cytoplasm of TGR. There were no signs of hypertrophy, inflammation, fibrosis or cardiac failure in these rats. This may suggest that exon(2-9)renin is rather protective than detrimental. More detailed investigations are needed to establish the function of exon(2-9)renin in the heart in various physiological and pathophysiological contexts. Since in adult rat hearts only exon(1A-9)renin transcripts are detected, the new transgenic model can be used to study the functions of the encoded exon(2-9)renin protein in the heart *in vivo*.

### Exon(2-9)renin and the adrenal gland

In the rat adrenal gland both the exon(1–9)renin and the exon(1A-9) renin transcripts are expressed. It has been suggested that there is a functionally active adrenal RAS in the zona glomerulosa. This assumption was based on the fact that under some conditions, such as potassium load or bilateral nephrectomy, aldosterone levels correlate better with adrenal renin levels than with circulating renin levels [22–24]. The functional significance of intra-adrenal renin has been demonstrated *in vitro* and *in vivo*(for review see) [24, 25]. Thus, in adrenal explant cultures or adrenal monolayers the potassium-stimulated aldosterone production is reduced by ACE inhibitors or AT1 receptor blockers *in vitro*[26–29]. *In vivo*, the AT1 receptor antagonist losartan inhibits aldosterone production after bilateral nephrectomy [30], a procedure which eliminates circulating renin, but increases renin in the adrenal cortex [22]. There is now considerable evidence that there is a local secretory renin system in the adrenal cortex, which regulates aldosterone production [24, 25, 31].

Indirect evidence suggests that the adrenal cortex may harbour an intracellular renin system in addition to the secretory system. Like the secretory system, the intracellular renin system may stimulate aldosterone production. Renin was detected immunhisto-chemically and enzymatically within adrenocortical mitochondria [12, 13]. Further, exon(2-9)renin but not exon(1-9)renin or active renin was imported into isolated mitochondria *in vitro*[8]. Also, bilateral nephrectomy markedly increased mitochondrial renin activity (about 20-fold) as well as aldosterone production.

The present study supports the hypothesis that intracellular exon(2-9)renin may be functionally active. We demonstrate that the expression of exon(2-9)renin increases plasma renin-independent aldosterone production in three out of four transgenic lines. The fourth line, which did not exhibit elevated adrenal renin levels in the cytosol or high-density fraction, also did not exhibit increased plasma renin-independent aldosterone production.

### Exon(2-9) and blood pressure regulation

The circulating RAS, determined by secretory exon(1-9)renin, is known to increase blood pressure. In the present study, overexpression of non-secretory, cytoplasmatic exon(2-9)renin was not associated with an increase in systolic blood pressure when compared with WT controls. Recently, astrocyte-specific overexpression of human(2-9)renin together with human angiotensinogen has been reported to lead to increased blood pressure in double transgenic mice (32). The discrepant findings between this study (32) and ours with respect to blood pressure may be explained by the different species (mice *versus* rat) or different constructs (astrocyte specific promoter *versus* non specific promoter) used. Furthermore, plasma renin activity was (unexpectedly) increased in the double transgenic mouse model whereas it was normal in our TGR.

To date, little information is available on the potential functions of cytoplasmatic exon(2-9)renin. Like conventional renin, exon(2-9)renin may generate ANGI from angiotensinogen. Intracellular actions of ANGII have been proposed as early as 1971 by Robertson *et al.*[33] and later by Re and others [34, 35]. In these studies, nuclear [33] or mitochondrial [33, 34] localizations of ANGs were demonstrated. Furthermore, intracytoplasmatically applied ANGII produced effects without the need to bind ANG receptors from the extracellular space. Thus, instillation of renin or ANGI in cardiac cells altered intracellular calcium levels, ion fluxes and intercellular conduction [35–38].

For intracellular ANG production to occur, cytoplasmatic exon(2-9)renin must gain access to angiotensinogen. Although this has not been directly demonstrated, an experimental model is available that supports intracellular ANG production from cytoplasmatic angiotensinogen: In rat hepatoma cells expressing only exon(1A-9)renin, overexpression of cytoplasmatic angiotensinogen increased mitosis and proliferation rates [39]. Furthermore, cytoplasmatic exon(2-9)renin may act on additional targets other than angiotensinogen. For instance, a cytoplasmat-ically localized renin-binding protein has been described [40]. This renin-binding protein is identical to the enzyme N-acetyl-D-glucosamine 2-epimerase [41], inhibits renin activity [42] and is inhibited by renin [43]. More recently, a renin receptor has been found with partially intracellular location [44, 45].

Taken together, the results obtained in four different lines of exon(2-9)renin TGR collectively demonstrate that the protein encoded by the exon(1A-9)renin transcript (1) is translated into a functionally active intracellular protein; (2) is targeted to the cytoplasm and a high-density fraction rather than being sorted to the secretory pathways and (3) is functionally active, reg-ulating aldosterone production. The CX-(exon2-9)renin transgenic rat appears to be a useful model to study the role and the mechanisms of action of cytoplasmatic renin derived from exon(1A-9) transcripts.
